# Supplementary material for: “Single Knot–Single Running Suture” Vesicourethral Anastomosis with Posterior Musculofascial Reconstruction during Robot-Assisted Radical Prostatectomy: A Step-by-Step Guide of Surgical Technique
Source: J Pers Med. 2023 Jun 29;13(7):1072. doi: 10.3390/jpm13071072 (PMC10381871; doi:10.3390/jpm13071072)
Supplement: Supplementary file 1 [file jpm-13-01072-s001.zip › jpm-2467128-supplementary.pdf]

Supplementary Table 1. Covariates comparison after 1:1 propensity score matching between Van Van Velthoven anastomosis modified sec. Gallucci (VV-G) vs conventional Van Velthoven anastomosis (VV-STD) during RARP.

Characteristic	OVERALL<br>N = 80	VV-GALLUCCI[1]<br>n = 40 (50%)	VV-STD[1]<br>n = 40 (50%)	p-value[2]
**BMI** *(kg/m2)*	26.7 (25.2, 29.4)	25.9 (24.7, 29.2)	27.1 (25.6, 30.1)	0.2
**Prostate volume** *(cc)*	40 (30, 56)	37 (30, 47)	42 (30, 60)	0.2
**Charlson Comorbidity Index**	3.00 (2.00, 4.00)	3.00 (2.00, 4.00)	2.50 (2.00, 4.00)	0.3
**Nerve-Sparing**				0.8
No	42 (52.5%)	21 (52.5%)	21 (52.5%)	
Monolateral	14 (17.5%)	8 (20.0%)	6 (15.0%)	
Bilateral	24 (30.0%)	11 (27.5%)	13 (32.5%)	
**ePLND**	30 (37.5%)	15 (37.5%)	15 (37.5%)	>0.9

[1]Median (IQR); n (%)

[2]Wilcoxon rank sum test; Pearson's Chi-square test
